# MicroRNA-8073: Tumor suppressor and potential therapeutic treatment

**DOI:** 10.1371/journal.pone.0209750

**Published:** 2018-12-27

**Authors:** Atsuko Mizoguchi, Aiko Takayama, Taiga Arai, Junpei Kawauchi, Hiroko Sudo

**Affiliations:** New Frontiers Research Laboratories, Toray Industries, Inc., Kamakura, Kanagawa, Japan; University of South Alabama Mitchell Cancer Institute, UNITED STATES

## Abstract

The comprehensive screening of intracellular and extracellular microRNAs was performed to identify novel tumor suppressors. We found that miR-8073 was present in exosome and predominantly exported from colorectal cancer cells. Treatment with a synthetic miR-8073 mimic resulted in a dramatic decrease in the proliferation of various types of cancer cells, which was not observed in similarly treated normal cells. As little is known about the biological functions of miR-8073, its target mRNAs were analyzed by both mRNA expression and *in silico* sequence analyses, leading to five probable target candidates (*FOXM1*, *MBD3*, *CCND1*, *KLK10*, and *CASP2*) that enhance survival during the regulation of the cell cycle, cell proliferation, and apoptosis. We experimentally confirmed that miR-8073 binds the 3’-UTR of each of these mRNA target candidates and that the introduction of a synthetic miR-8073 mimic into cancer cells reduced levels of protein expression. Finally, the antiproliferative effects of miR-8073 were validated *in vivo*: the subcutaneous injection of a synthetic miR-8073 mimic suppressed colorectal tumor volume to 43% in tumor-bearing xenografted mice. These results suggest that because miR-8073 binds, and thus reduces the levels of, these oncogenic targets, cancer cells must actively downregulate miR-8073 as a survival mechanism. The introduction of miR-8073 into tumors could thus inhibit tumor growth, indicating its great potential for cancer therapeutics.

## Introduction

MicroRNAs are a class of endogenous, small, noncoding RNAs of approximately 22 nucleotides in length. They regulate the translation of their target genes by binding to complementary sequences in the 3’-untranslated regions (3’-UTRs) of the target mRNAs [1]. It is thought that microRNAs control more than 60% of human genes that encode proteins involved in a series of cell functions, including cell proliferation, apoptosis, and differentiation [2].

MicroRNAs circulate stably in the peripheral blood, because they are enclosed in exosomes or form complexes with Ago proteins or high-density lipoproteins. Based on the fair amount of microRNAs present in human peripheral blood and their physical robustness, microRNAs may serve as potential biomarkers for various diseases, including cancer. We have constructed microRNA diagnostic indices that accurately distinguish healthy volunteers or patients with benign diseases from pancreatobiliary cancer [3] or breast cancer patients [4]. Others have identified circulating microRNAs that are indicative of colorectal cancer [5–8]. Whether the release of these microRNAs into the peripheral blood is causative or consequential to cancer development is unknown, and many of the proposed theories require further clarification.

Beyond their diagnostic potential, microRNAs have been recently touted as a new type of cancer treatment. For instance, miR-1 [9], miR-21 [10], miR-125a-5p [11], and miR-34a-5p [12] have been reported to play significant roles in cancer cell proliferation.

In this study, we screened extracellularly and intracellularly localized microRNAs from cancer cells in order to search novel tumor suppressors; we found that miR-8073 was predominantly exported from cancer cells and elicited antiproliferative effects both *in vitro* and *in vivo* when administered. We also confirmed its molecular mechanism and demonstrated its potential use as a cancer treatment.

## Materials and methods

### Cell culture

The following human cell lines were obtained from the American Type Culture Collection (Manassas, VA USA): HCT116 and HT29 (colon cancer), MCF7 (breast cancer), Panc-1 and Panc10.05 (pancreatic cancer), A549 (lung cancer), HEK293T (embryonic kidney), and 184B5 (mammary gland epithelium). The human lung microvascular endothelial cell line HMVEC-L and the mammary epithelial cell line HMEC were obtained from Lonza (Basel, Switzerland). The human colonic epithelial cell line HCOEpiC was obtained from ScienCell Research Laboratories (San Diego, CA USA).

HT29, Panc-1, and HEK293T cells were maintained in Dulbecco’s modified Eagle medium (Nacalai Tesque, Japan) supplemented with 10% fetal bovine serum and antibiotics at 37°C in 5% CO_2_. MCF7, Panc10.05, and A549 cells were maintained in RPMI 1640 medium (Nacalai Tesque) supplemented with 10% fetal bovine serum and antibiotics at 37°C in 5% CO_2_. HCT116 cells were maintained in McCoy’s 5A medium (Thermo Fisher Scientific, Waltham, MA, USA) containing 10% fetal bovine serum and antibiotics at 37°C in 5% CO_2_. HCOEpiCs were maintained in colonic epithelial cell medium (ScienCell) containing a 1% penicillin-streptomycin solution at 37°C in 5% CO_2_. HMVECs were maintained in EGM-2 medium (Lonza) containing EGM-2MV SingleQuots at 37°C in 5% CO_2_. The normal breast cell line 184B5 and HMECs were maintained in MEBM medium (Lonza) supplemented with bovine pituitary extract, hydrocortisone, hEGF, and insulin at 37°C in 5% CO_2_.

### Intracellular, extracellular, and exosomal microRNA extraction from cultured cells

Cells were grown in 10-cm plates for 48 hours beforehand, then cells and culture supernatant were collected. The medium was replaced with either “advanced DMEM” (Thermo Fisher Scientific) or RPMI containing an antibiotic-antimycotic mixture and 2 mM L-glutamine (not containing fetal bovine serum), and incubated for 48 hours. Approximately 6 × 10^4^ cells and 1.5 mL cell culture supernatant (into which extracellular particles such as exosomes were released) were collected.

Exosomes were prepared by further extraction from the cell culture supernatant; cells and cell debris were removed by centrifugation at 2,000 × *g* for 10 minutes at 4°C and filtration, followed by further centrifugation at 110,000 × *g* for 70 minutes at 4°C. The pellets were washed and resuspended in 11 mL phosphate-buffered saline, and centrifuged again at 110,000 × *g* for 70 minutes at 4°C [13]. Finally, the pellet (exosomes) was resuspended in 300 μL phosphate-buffered saline.

Total RNA derived from the cell culture supernatant or exosomes was extracted using the 3D-Gene RNA extraction reagent (Toray Industries, Inc., Japan), whereas total RNA derived from cells was extracted using the miRNeasy Mini kit (QIAGEN, Hilden, Germany, catalog #217004). (dx.doi.org/10.17504/protocols.io.vu3e6yn)

### Cell proliferation, apoptosis, and mRNA extraction of microRNA-transfected cells

Cells were grown on 96-well plates, and 1.0 × 10^3^ cells per well were transfected with either a synthetic hsa-miR-8073 mimic (Thermo Fisher Scientific, mirVana miRNA mimic, catalog #4464066, Assay ID; MC29125) or a microRNA-negative control sequence (Thermo Fisher Scientific, mirVana miRNA Mimic, Negative Control #1 catalog #4464058) at a concentration of 0.03–30 nM using Lipofectamine RNAiMAX Transfection Reagent (Thermo Fisher Scientific, catalog #13778150), according to the manufacturer’s protocol. To examine transfection efficiency, total microRNA was isolated from the transfected cells using the miRNeasy Mini kit (QIAGEN) and quantified using the Taqman microRNA assay (Thermo Fisher Scientific, catalog# 440886, Assay ID;466465_mat). PCR experiments were conducted in triplicate, with the following thermal conditions: 95°C for 10 minutes, followed by 95°C for 15 seconds for 40 cycles and 60°C for 1 minutes. When the miR-8073 expression signal in the miR-8073-treated cells was greater than that in the mock-treated cells, the transfection was considered successful.

After transfection, cell viability was measured using the Celltiter-Glo 2.0 luminescent cell viability assay reagent (Promega, Madison, WI, USA, catalog #G9241). Luminescence obtained from this assay was read using a Veritas microplate luminometer. Cell viability was statistically analyzed using *P* values generated from the Student’s *t* test.

Apoptosis was evaluated by measuring the activity of caspase-3 and -7 using the Caspase-Glo 3/7 assay systems (Promega, catalog #G8090).

For mRNA expression profiling, five days after transfection with either the miR-8073 mimic, a microRNA-negative control sequence, or a blank, total RNA was extracted from 2 × 10^5^ HCT116 cells using the miRNeasy Mini kit (QIAGEN). The quantity, length, and integrity of the obtained RNA were assessed using the NanoDrop (Thermo Fisher Scientific) and Agilent 2100 (Agilent Technologies, Santa Clara, CA) instruments. The linear amplification of 0.5 μg total RNA was performed using the Ambion amino allyl MessageAmp II aRNA amplification kit (Thermo Fisher Scientific, catalog# AM1751).

### MicroRNA and mRNA expression analysis from cancer and normal cells

Comprehensive microRNA expression analysis was performed using the 3D-Gene human microRNA oligo chip (Toray Industries, Inc., Japan), which was designed to detect 2,565 microRNAs registered in miRBase release 21 (http://www.mirbase.org/). Similarly, comprehensive mRNA expression analysis was performed using the 3D-Gene human oligo chip 25K manual (v. 2.01). The microarray data signals were treated similarly as previously described [3]. To normalize signals across the different microarrays, quantile normalization was applied. Numeric analyses were performed using R v. 3.1.2 (R foundation for Statistical Computing, http://www.R-project.org). All microarray data from this study are in agreement with the Minimum Information about a Microarray Experiment standard and are publicly available through the Gene Expression Omnibus database (http://www.ncbi.nlm.nih.gov/projects/geo/) under accession number GSE114318.

### Luciferase reporter assay to confirm microRNA binding to target genes

For luciferase reporter experiments, 831 bp of human FoxM1 3’-UTR segment, 969bp of CCND1 3'-UTR segment, 1945bp of KLK10 3'-UTR segment, 1045bp of MBD3 3'-UTR regions or 2606bp of CASP2 3'-UTR segment were generated by direct PCR amplification from human genomic DNA. The gel-purified PCR fragments were cloned into the SpeI/MulI backbone of the pMIR-REPORT Luciferase miRNA Expression Reporter

Vector (Thermo Fisher Scientific, catalog #AM5795). To create a full-length five types of gene 3′ UTR reporter, the following primers were used to amplify the 3’ UTR of genes from a human genomic DNA: FOXM1; 5′- AGACTAGTCCAAGGCTCAGTGCACCCCA

AGCCTC -3′ and 5′-ATACGCGTTGGCTCTTTGCAAAGCTGAGGGGCAAG-3′, CCND1; 5’-AGACTAGTCACAGCTGTAGTGGGGTTCTAGGCA -3’ and

5’- ATACGCGTTGTGTGTTTAAATCAAGGGGAGATTGC -3’,

KLK10; 5’- AGACTAGTAGATGTTATGCTCCTGCTGATCCAG-3’ and

5’-ATACGCGTACTCCTATAGCCTGGGCAATTCAG-3’,

MBD3; 5’- AGACTAGTCTCCTTGAGACTGGAGAGCAGCCAGCA -3’ and

5’- TACGCGTACCAACCTCAGGAAGACGTGGTCCCC -3’,

CASP2; 5’- AGACTAGTACCTCCCCATCATCCACGCCAAGTG -3’ and

5’- ATACGCGTGAGCATTTATTTGGCACCCGATGGCAATAC-3’.

The constructs were sequenced to ensure accuracy of the cloning procedure. HCT116 cells and HEK293 cells (2 × 10^5^ per well) were seeded in 12-well plates and incubated for one day before transfection. Five types of pMIR-REPORT luciferase reporter vector containing the FOXM1 3’-UTR, the CCND1 3’-UTR, the KLK10 3’-UTR, the MBD3 3’-UTR or the CASP2 3’-UTR, pMIR-REPORT luciferase vector with no insert, the pMIR-REPORT β-gal control plasmid were co-transfected with either a synthetic miR-8073 mimic (Thermo Fisher Scientific, mirVana miRNA Mimic, Assay ID; MC29125) or a microRNA-negative control sequence (Thermo Fisher Scientific, mirVanamiRNA Mimic, Negative Control #1) into cells using Lipofectamine RNAiMAX (Thermo fisher scientific). Twenty-four hours after transfection, luciferase activity was quantified using the dual-luciferase assay reporter system (Promega, catalog #E1910) and activities were normalized to β-galactosidase activity and the relative ratios of firefly to Renilla activity were measured. All experiments were performed in triplicate.

### Western blot analysis

One day after microRNA transfection, cells were harvested and lysed in RIPA lysis buffer. The protein concentrations of the cell lysates were quantified using a bicinchoninic acid kit, and equal amounts of proteins were separated by SDS-PAGE and transferred to polyvinylidene fluoride membranes (Merck, Darmstadt, Germany). The membranes were blocked in 5% non-fat dry milk at room temperature for 1 hour and incubated overnight at 4°C with a 1:1,000 dilution (in PBST) of specific primary antibody: anti-FOXM1 (Santa Cruz, Dallas, TX, USA, sc-376471, G-5, mouse monoclonal IgG2b kappa light chain), anti-MBD3 (AbCam, Cambridge, England, ab157464, rabbit monoclonal), anti-CCND1 (AbCam ab134175, rabbit monoclonal), anti-KLK10 (AbCam ab55623, mouse mononclonal), or anti-CASP2 (AbCam ab32021, rabbit monoclonal). The membranes were then incubated with goat anti-mouse IgG (or goat anti-rabbit IgG) conjugated to horseradish peroxidase secondary antibody at a 1:1,000 dilution (Cell Signaling Technology Inc., MA, USA) for 2 hour. The proteins were visualized using ECL reagents (Amersham Biosciences, Sweden). The density of the bands was quantified by ImageQuant TL software (v. 8.1, GE Healthcare, Little Chalfont, England), and the obtained numerical values were normalized to the densitometric value of β-actin in each sample.

### *In silico* analysis of microRNA targets

MicroRNA targets were first predicted through the following publicly available databases: TargetScan release 6.2 (http://www.targetscan.org/vert_71/), miRDB release 5.0 (http://www.mirdb.org/download.html), and microT release v. 5.0

(http://diana.imis.athena-innovation.gr/DianaTools/index.php?r=microT_CDS/index). The obtained target candidates were then selected based on the signaling pathway analysis performed using Metacore software

(https://portal.genego.com/cgi/data_manager.cgi) (Thomson Reuters, NY, USA).

### Administration of a synthetic miR-8073 mimic to tumor-bearing xenografted mice

Animal experiments were approved by Toray Industries’ committee for Ethics in Animal Experimentation (AC2016-64), and the experiments were conducted in accordance with the guidelines for animal experiments. HCT116 cells (5 × 10^6^ cells) were resuspended in a 1:1 mixture of serum free-RPMI 1640 medium and Matrigel (BD Biosciences, Franklin Lakes, NJ, USA) in a total volume of 0.1 mL and subcutaneously inoculated on the backs of female 6-week-old BALB/c nude mice (Charles River Laboratories, Wilmington, MA, USA). The tumor size was monitored twice per week by measuring its length and width with calipers, and tumor volumes were calculated using the following formula: [(major axis) × (minor axis)]^2^ × 0.5. When the tumor volume reached approximately 50–100 mm^3^ two days after inoculation, the mice were randomized into two treatment groups: five mice for the microRNA-negative control sequence (Thermo Fisher Scientific, mirVana miRNA Mimic, Negative Control #1 catalog#4464061) and five mice for the miR-8073 mimic (Thermo Fisher Scientific, mirVana miRNA mimic, catalog#4464070, Assay ID; MC29125). Two nmol of the miR-8073 mimic or negative control were prepared with 0.5% AteloGene Local Use (Koken, Japan); 50 μL of these mixtures were administered under the skin adjacent to the tumor of tumor-bearing mice on the timing of days 2, 4, and 7 after cell inoculation.

### Statistical analysis

Differences between subgroups were tested by Student’s t-test. A P value of < 0.05 was considered statistically significant.

## Results

### Screening of tumor suppressor microRNAs in cultured cancer cells identifies miR-8073

**Fig 1:**
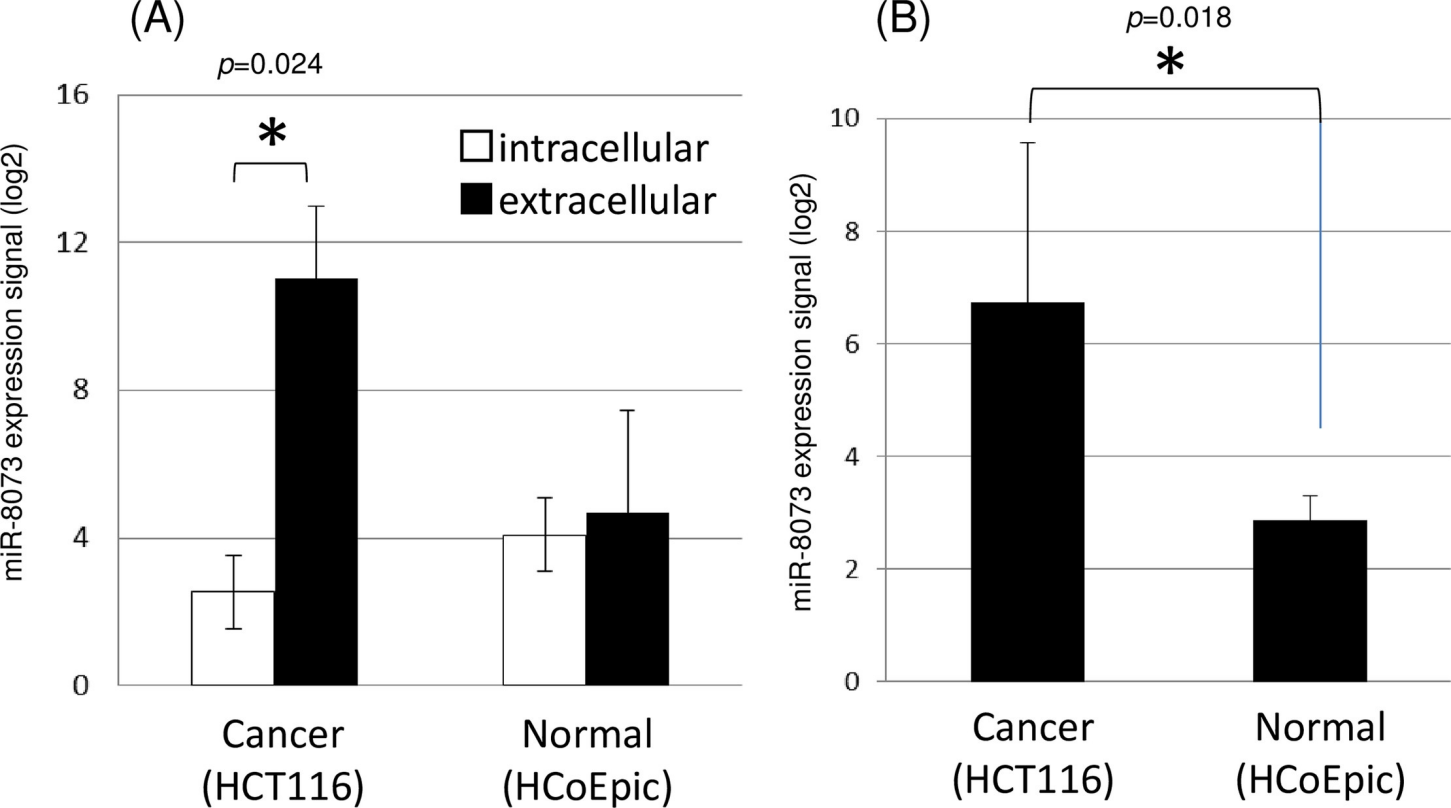
**Intracellular, extracellular (A) or exosomal (B) miR-8073 expression in 6x10**^**4**^ **colorectal cancer cell HCT116 and normal cell HCoEpic.** In Fig 1B, exosomes that were extracted from the same amount of the cell cultured supernatant used in Fig 1A, were assayed for microRNA expression profiling. MiR-8073 was one of microRNAs that was most exported to extracellular space in the cancer cells but much less in the normal cells. The error bar indicates standard deviation. The star indicates p<0.05 in student’s t-test.

We first hypothesized that tumor suppressor microRNAs should be present in lesser amounts inside versus outside of cancer cells due to an active export mechanism. Using HCT116 and Panc-1 cells, we performed comprehensive microRNA expression profiling analyses of the cellular content, the culture supernatant (all extracellular materials), and the exosomal fraction of the supernatant from cells grown on the same 10-cm dish. Out of 2,565 microRNAs registered in the miRBase, 970 microRNAs were present at lower levels inside the cells versus outside of the cells. Among these microRNAs, six (miR-8073, miR-1290, miR-642a-3p, miR-1249-5p, miR-4792, and miR-4732-5p) had a 2^6^ times higher expression signal in the supernatant than in the cell (S1 Fig). On the other hand, known tumor suppressor microRNAs, miR-1 [9], miR-21 [10], miR-34a-5p [11], and miR-125a-3p [12], did not show differences in expression levels between the intra- and extracellular regions in this study (S1 Fig). Out of these six microRNAs, miR-8073 did not show a dramatic differential expression in normal colorectal cells (HCoEpiC) in contrast to that observed in cancer cells (Fig 1A). Interestingly, miR-221-5p, which has the same seed sequence as miR-8073 and is reported to be involved in cell proliferation and vascularization / angiogenesis [14], was not exported into the extracellular space by the cancer cells (S1 Fig).

Additionally, to determine which form of the microRNA was exported from the cancer cells, we extracted exosomes from the cell culture supernatant and measured the expression of exosomal miR-8073 (Fig 1B, S2 Fig). In normal cells, the miR-8073 signal ratio of the exosomal to the whole supernatant was approximately 30%; however, in cancer cells, the signal ratio eminently increased up to 50% (Fig 1B). This result indicates that cancer cells recruit exosomes as cargo more actively than do normal cells in order to export miR-8073 from the intracellular space.

### The miR-8073 mimic inhibits proliferation of cancer cells but not of normal cells

**Fig 2:**
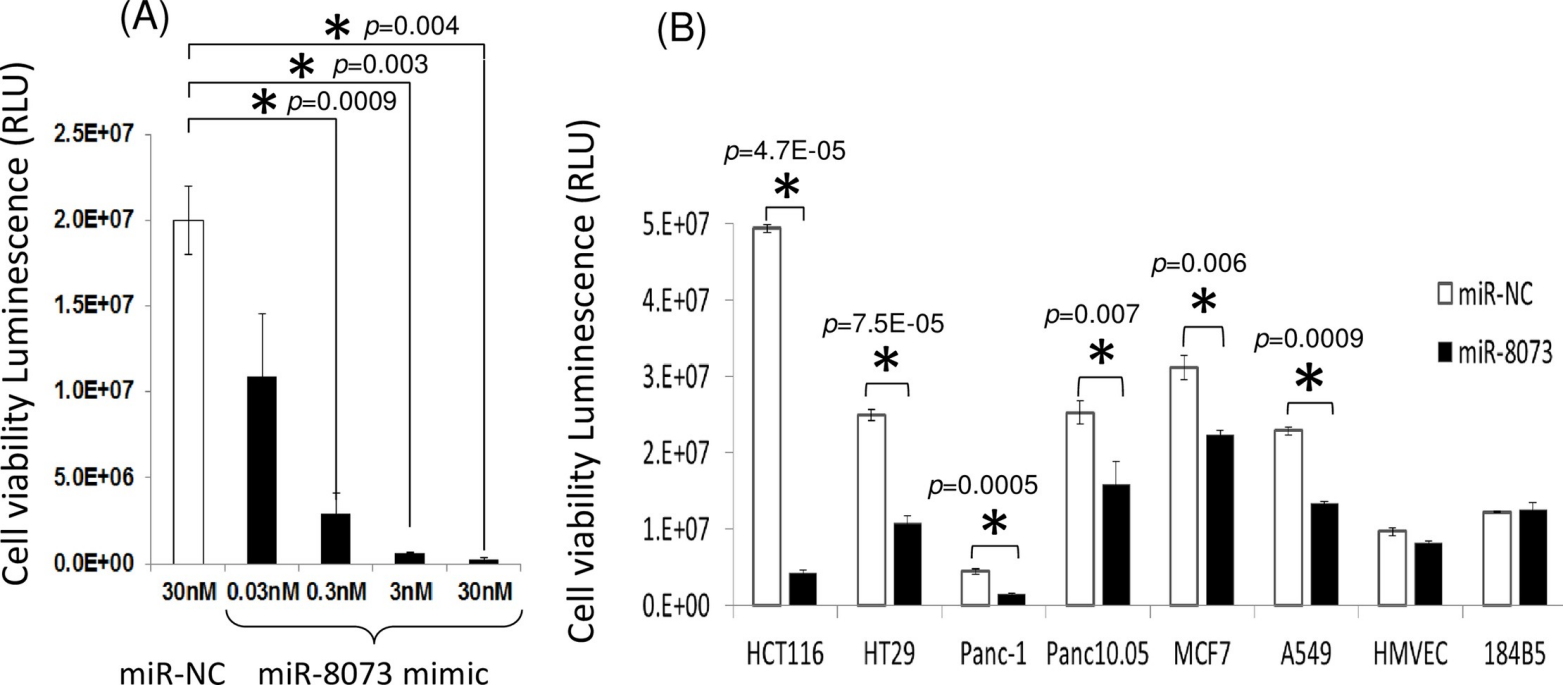
Viability of human colorectal cancer cells HCT116, and other types of cancer cells or normal cells. Viability of human colorectal cancer cells HCT116, and (B) other types of cancer cells or normal cells at fifth day from transfection with miR-8073 mimic or negative control sequence (miR-NC). Concentrations of the synthetic nucleic acids used for the transfection were either 0.03nM to 30nM (A) or 30nM (B). In Fig 2B, cells tested were colorectal (HCT116, HT29), pancreatic (Panc-1, Panc0.05), breast (MCF7), lung (A549) cancer cells, and normal cells derived from microvascular endothelium (HMVEC) or mammary epithelium (184B5). The error bar indicates standard deviation. The star indicates p<0.05 in student’s t-test.

To examine the effects of miR-8073 on cellular proliferation, a synthetic miR-8073 mimic was transfected into HCT116 cells. Five days post-transfection, the miR-8073 mimic (30 nM) reduced cancer cell viability to 4% of the levels in cells transfected with a negative control sequence at the same concentration (Fig 2A, S3 Fig). Even at a concentration of 0.3 nM, the miR-8073 mimic caused a greater than 50% reduction in cancer cell viability. Similar inhibitory effects of miR-8073 on cell proliferation were also confirmed in other types of cancer cells: the colon cancer cell line HT29, pancreatic cancer cell lines Panc-1 and Panc10.05, the breast cancer cell line MCF7, and the lung cancer cell line A549, but not in normal HMVECs or 184B5 cells (Fig 2B). On the other hand, a miR-8073 antisense sequence had no effects on any cancer cells. Similarly, the known tumor suppressor miR-1 [9] also induced anti-proliferative effects in HCT116 cells, but this result could not be reproduced in four other cancer cell lines, including in A549 cells (S4 Fig).

**Fig 3:**
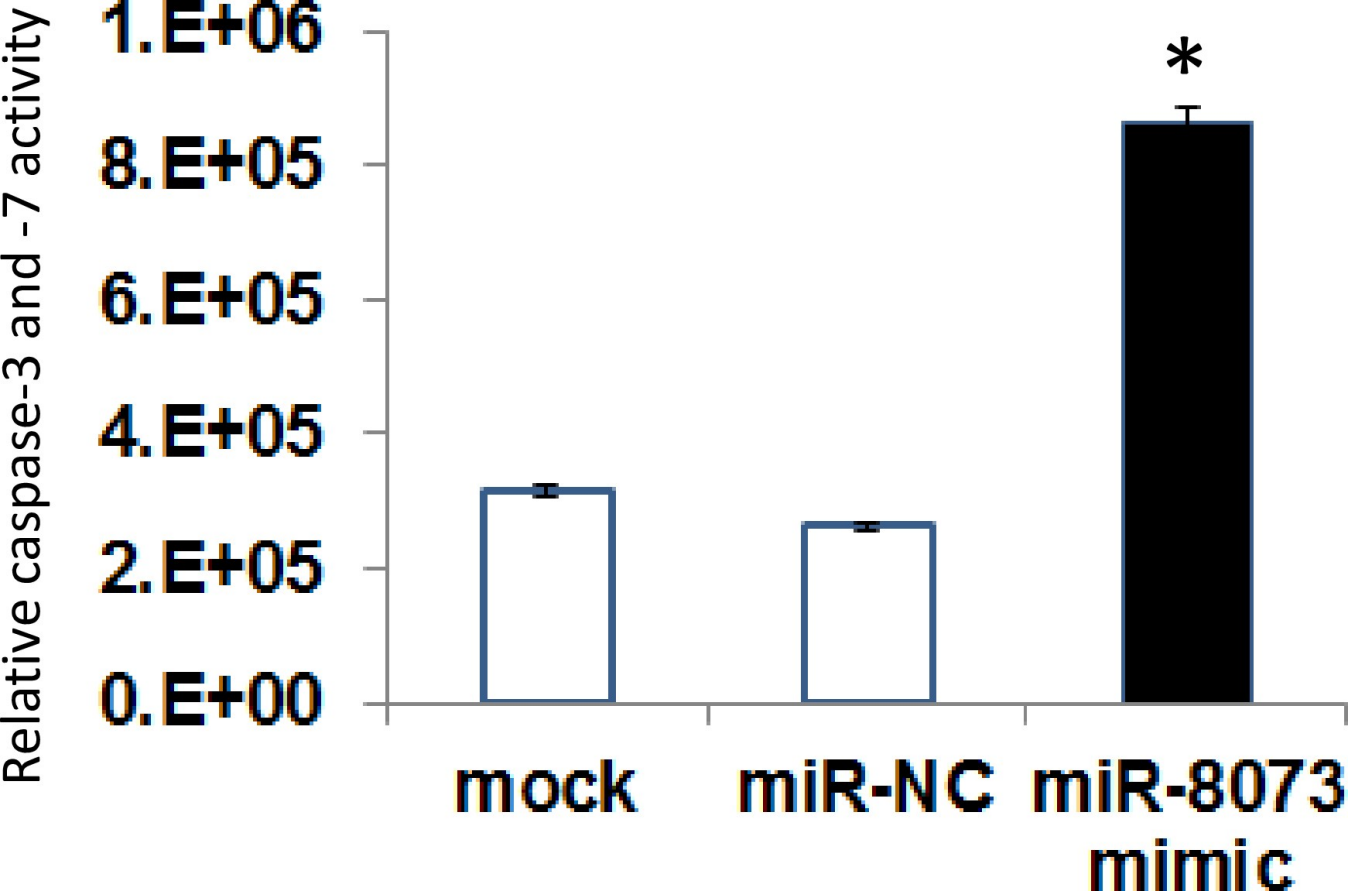
Apoptotic activity induced by miR-8073 mimic in colorectal cancer cell HCT116. Caspase-3/7 activity was measured after transfection for 72h, 30nM of miR-8073 mimic or a negative control sequence (miR-NC). The level of caspase-3 and caspase-7 activity in the mock treated cells was set at 100%. The error bar indicates the standard deviation.

We then examined whether the reduced viability of cancer cells induced by miR-8073 was associated with apoptosis. Three days after transfection of the miR-8073 mimic at 30 nM, we observed a 200% increase in the activities of caspase-3 and -7 proteins in colorectal cancer cells compared with mock-treated cells (Fig 3). These results suggest that miR-8073 can reduce cell viability in cancer cells via apoptosis.

### miR-8073 negatively regulates *FOXM1*, *CASP2*, *MBD3*, *KLK10*, and *CCND1* by directly targeting their 3’-UTRs, leading to suppression of protein expression

We demonstrated that cancer cells actively export miR-8073 from the intracellular space and that the overexpression of miR-8073 impairs cancer cell viability via apoptosis *in vitro*. Despite these astonishing findings, little is known about how miR-8073 triggers the signaling cascade and eventually causes such striking effects on cell proliferation and viability. We therefore searched for potential target genes of miR-8073 by comprehensive mRNA expression and *in silico* sequence prediction analyses of public databases.

In the former method, comprehensive mRNA expression profiling was performed on HCT116 cells one day after transfection with 30 nmol miR-8073 mimic, a microRNA-negative control sequence, or a blank, each assayed in triplicate using microarray. Differentially expressed genes were selected based on a comparison between the miR-8073 group and the negative control group, as well as a comparison between the miR-8073 group and the blank group, using the following statistical criteria: *P*-value less than 0.001 in an unpaired Student’s *t* test, fold change greater than 2.0, and a mean signal strength (log2) greater than 5 in any group. As a result, 60 mRNAs were identified as genes that decreased after the introduction of miR-8073 into the cancer cells (S1 Table).

In the latter method, we used publicly available microRNA target prediction tools, such as TargetScan, miRDB, and microT, to investigate potential targets of miR-8073. Interestingly, each database predicted somewhat unique target genes: 2,022 genes for TargetScan 6.2, 411 genes for miRDB, and 529 genes for microT. Among them, 106 genes were commonly drawn from all three database searches.

We then integrated the results of the two methods: 60 genes obtained from the mRNA expression analysis and 106 genes obtained from the *in-silico* database analyses, resulting in 21 genes that were identified using both methods (S1 Table).

For these 21 genes, enrichment analysis of gene ontology was performed using Metacore by GO Processes; five genes (*FOXM1*, *MBD3*, *CCND1*, *KLK10*, and *CASP2*) were determined to be related to each other by the regulation of the cell cycle. These genes were found to be associated with proliferation [15–18], cell cycle [19], carcinogenesis [20], DNA methylation [21], and apoptosis [22], respectively. Significantly decreased mRNA expression levels of these genes in miR-8073-treated HCT116 cells were reconfirmed by qRT-PCR (S5 Fig).

**Fig 4:**
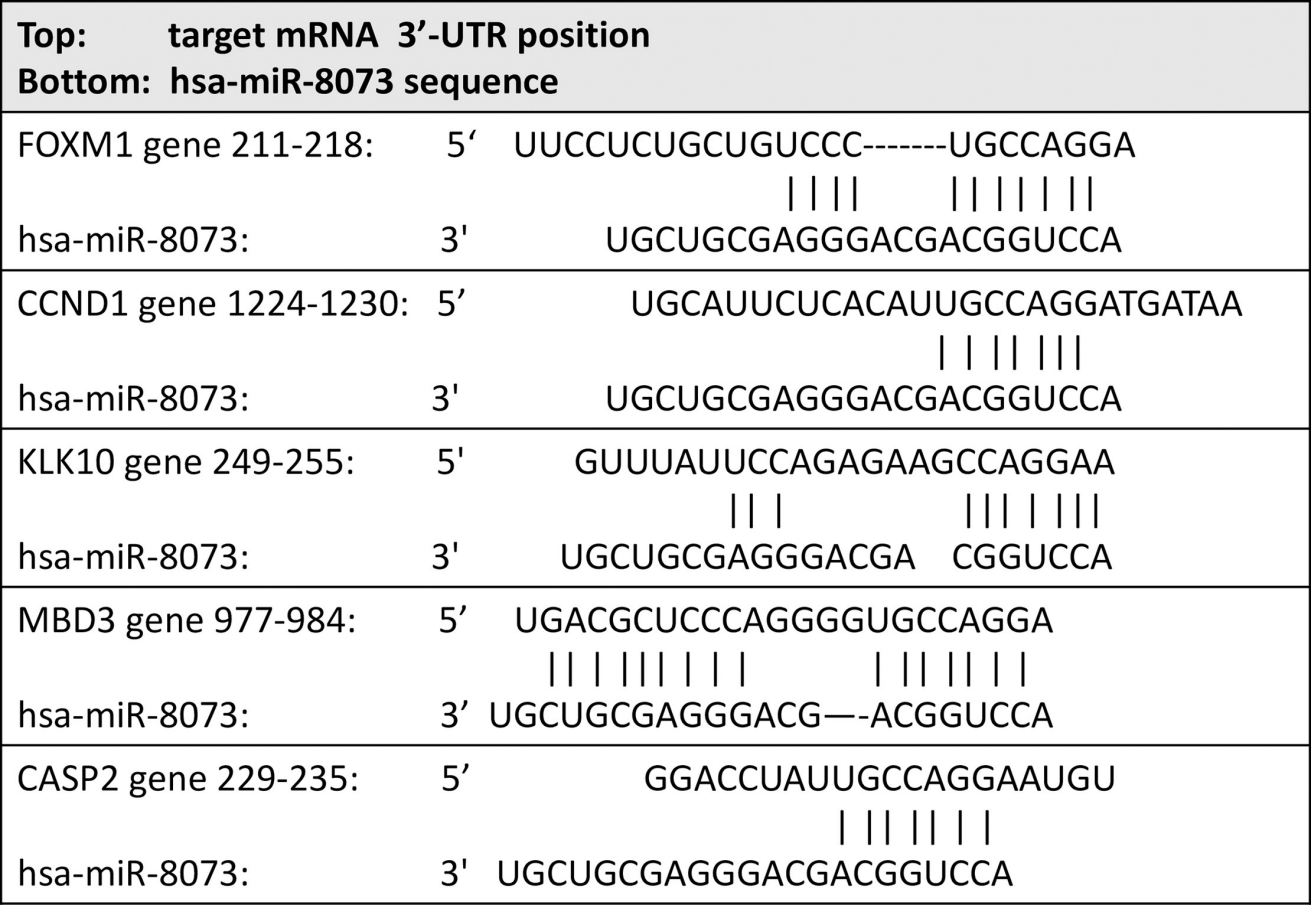
Potential mRNA targets of miR-8073 and their predicted binding sites.

**Fig 5:**
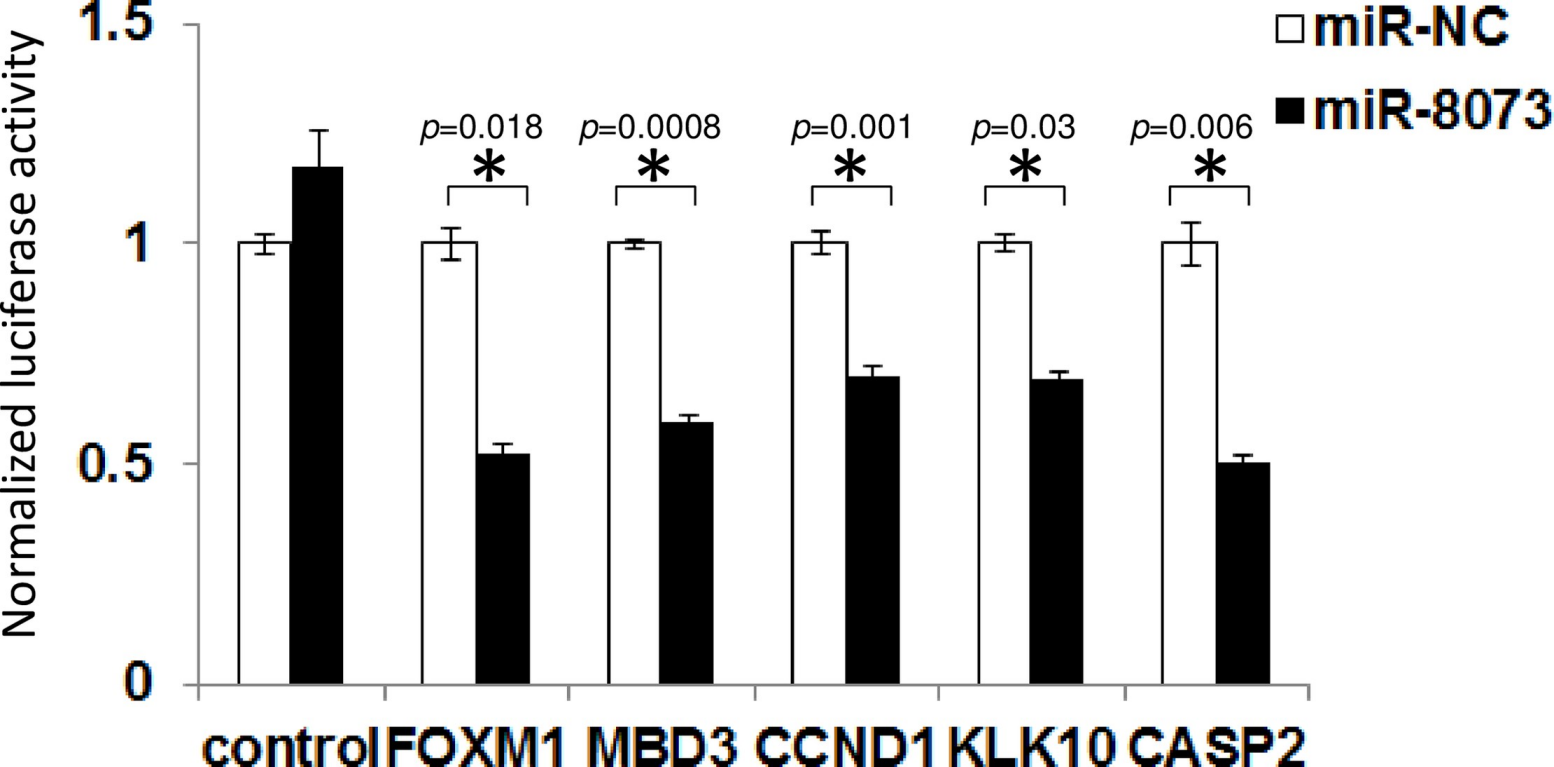
miR-8073 targeted FOXM1, MBD3, CCND1, KLK10 and CASP2 in HCT116 cells. Relative luciferase activity of HCT116 cells co-transfected with either 3nM of miR-8073 mimic or microRNA negative control (miR-NC) and with the luciferase reporter construct in which the 3’-UTR of each gene of interest was inserted. The control indicates the cells transfected with the same luciferase reporter plasmid without the added 3’UTRs, and the activity level of the control with miR-NC was set as 1.0. The error bars indicate the standard error of triplicate samples. The star indicates p<0.05 in student’s t-test.

**Fig 6:**
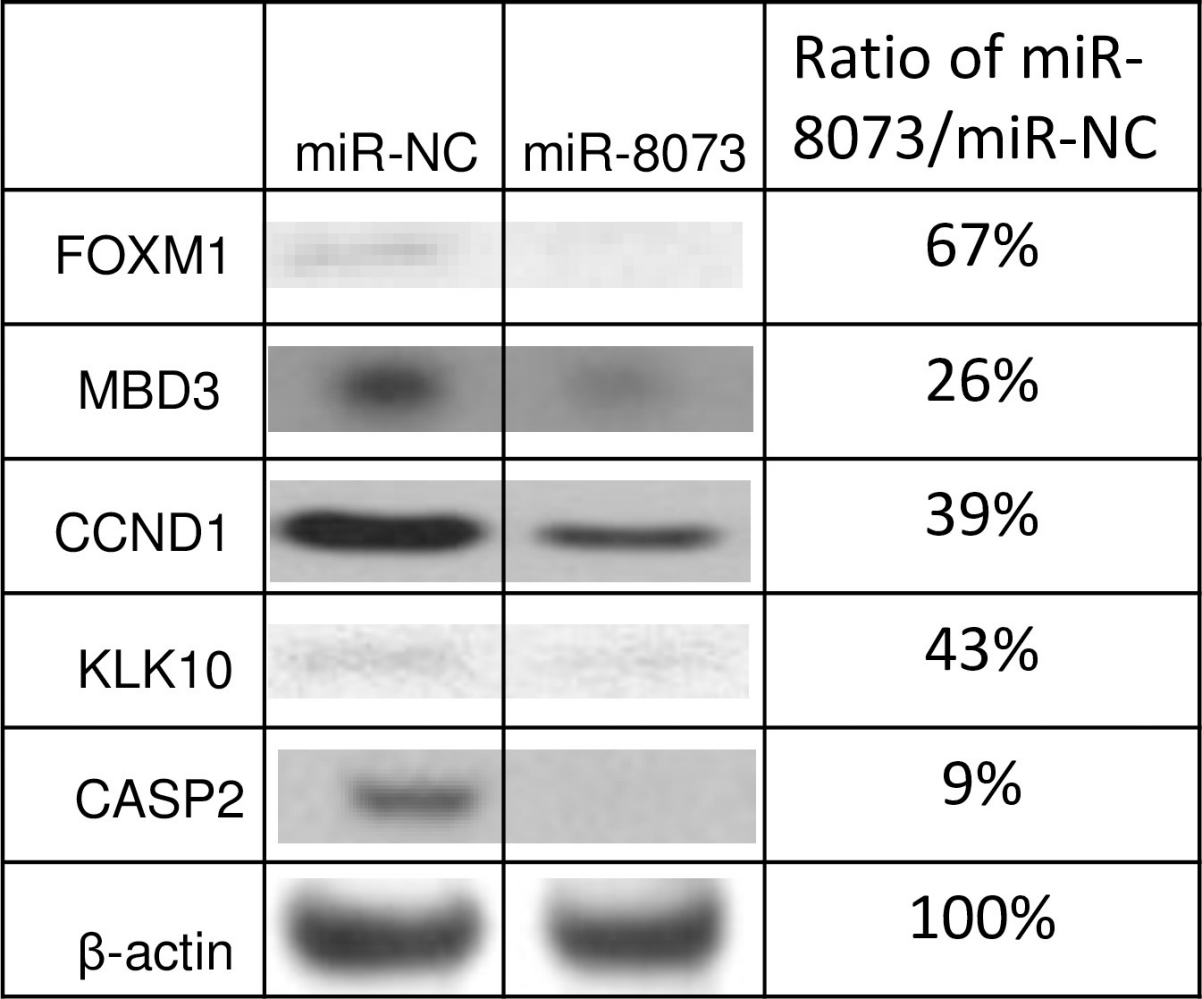
Western blot analysis of FOXM1, MBD3, CCND1, KLK10 and CASP2 expression in the HCT116 cells. Protein expression levels of FOXM1, MBD3, CCND1, KLK10 and CASP2 in HCT116 cells transfected with either 30nM of miR-8073 mimic or microRNA negative control (miR-NC) detected by Western blots. The images were numerated by software and ratios of the protein expression signal of miR-8073 treated cells over miR-NC treated cells were calculated.

To demonstrate the molecular association of miR-8073 and its target genes, the 3’-UTR sequences of these genes were investigated, and possible binding sites for miR-8073 were identified for each gene (Fig 4). To determine whether miR-8073 directly binds to these targets, reporter gene assays were performed; the 3’-UTR sequences of these target genes were inserted immediately downstream of a luciferase reporter gene and co-transfected with either miR-8073 or a negative control in HCT116 cells (Fig 5). Compared to cells treated with the negative control, miR-8073-overexpressing HCT116 cells showed a simultaneous reduction in the mRNA expression levels of FOXM1, MBD3, CCND1, KLK10, and CASP2 to 79%, 75%, 69%, 72%, and 58%, respectively (S5 Fig). The result confirms that miR-8073 interacts with the intact 3’-UTR sequences of these genes and regulates their expression level. Similar results were also obtained in HEK293 cells (S6 Fig), suggesting that these signaling pathways are not unique to specific cell lines. Furthermore, miR-8073 overexpression in HCT116 cells also reduced the protein expression levels of FOXM1, MBD3, CCND1, KLK10, and CASP2 to 67%, 26%, 39%, 43%, and 9% (Fig 6). These results strongly support the role of miR-8073 as a direct suppressor of these five genes and their corresponding proteins.

### Administration of the miR-8073 mimic suppresses colorectal tumor growth *in vivo*

**Fig 7:**
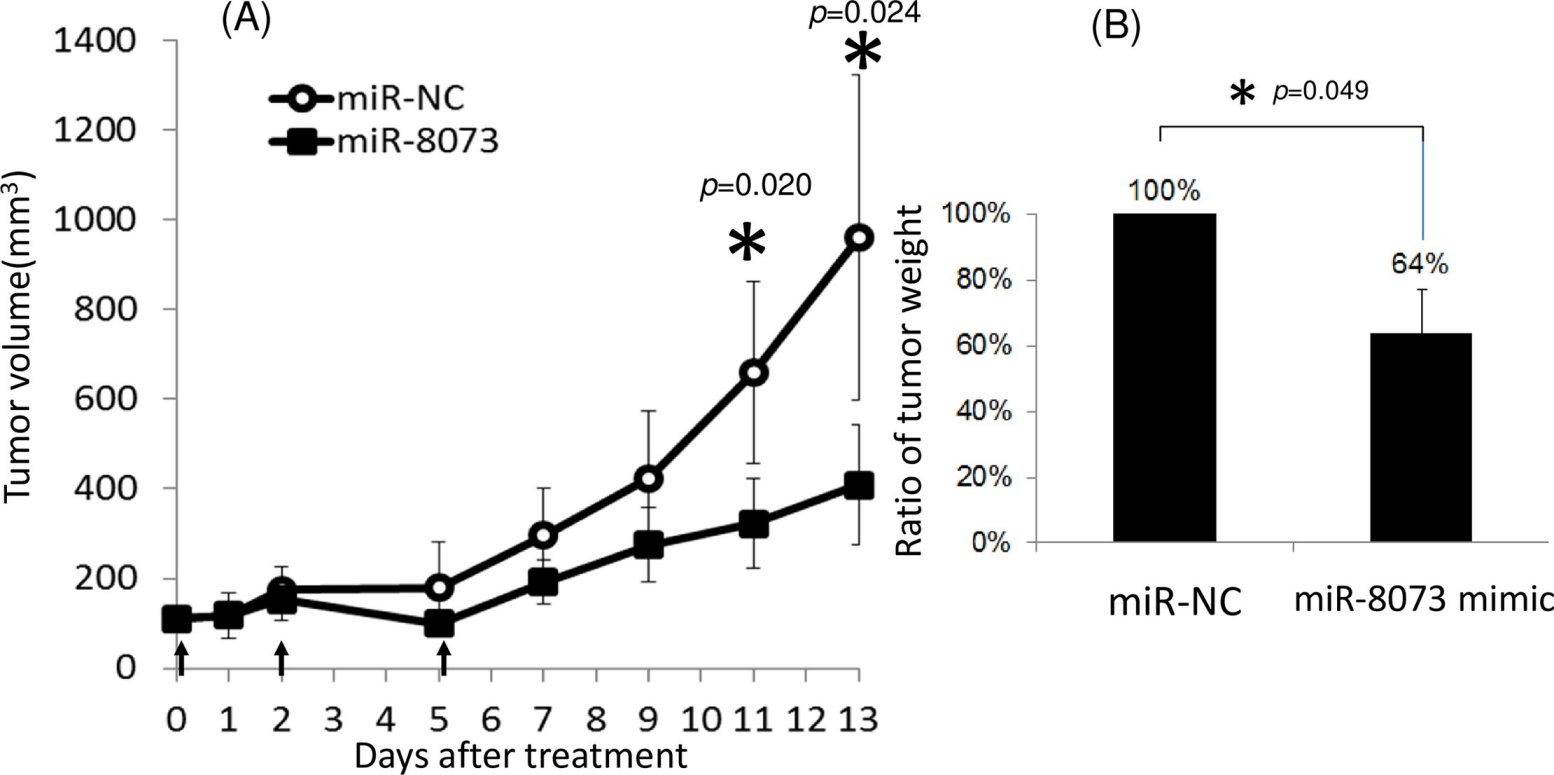
**Administration of miR-8073 mimic significantly reduced colorectal tumor volume (A) and weight (B) in xenografted mice**. BALB/c nude mice was transplanted with 5x10^6^ HCT116 cells two days before the treatment. The mice were treated with either 2nmol of miR-8073 mimic or microRNA negative control (miR-NC) mixed with atelocollagen at the day 0, 2, 5 (arrows). The tumor weight (B) was measured at the day 13. The error bars indicate the standard error in five mice per treatment group. The star indicates p<0.05 in student’s t-test.

Finally, we examined the tumor suppressor ability of miR-8073 on colorectal tumors in mice. Two nmol of the miR-8073 mimic or negative control sequence mixed with atelocollagen were subcutaneously administered on days 0, 2, and 5 to mice that had received a xenograft of HCT116 cells and subsequently developed tumors. By day 11, tumor growth was significantly suppressed in miR-8073-treated mice, and by day 13, the tumor volume was 43% and the tumor weight was 64% of the control mouse levels (Fig 7). These results demonstrate the suppressive role of miR-8073 on human colorectal tumor growth *in vivo*.

## Discussion

Extracellular microRNAs that are present in cell culture supernatants *in vitro* or in blood or urine samples in clinical settings have attracted the interest of many researchers. Such microRNAs are either enclosed in exosomes or form complexes with Ago proteins or high-density lipoproteins, and are thought to participate in cell-cell communication processes, such as cancer metastasis and progression [23], or in autocrine signal amplification, during which exosomes return to their secreted cells [24]. In this study, we established a unique hypothesis about extracellular microRNAs; some may be dispensable or rather deleterious to survival of individual cells, such that host cells actively export them into the extracellular space. If cases of cancer, these microRNAs may possess an antiproliferative function. We used this hypothesis to discover a novel tumor suppressor microRNA and elucidate its biological function in cancer biology.

Our comprehensive screening methods led to the identification of miR-8073, as it was actively exported to the extracellular space via exosomes by cancer cells. Treatment with a synthetic miR-8073 mimic resulted in a significant reduction in the viability of various cancer cells due to increased apoptosis, which was not observed in normal cells. A target analysis revealed that miR-8073 could bind at least five different mRNAs (FOXM1, MBD3, CCND1, KLK10, and CASP2), which are known to regulate cell proliferation, DNA methylation, cell cycle, carcinogenesis, and apoptosis, respectively. The binding of miR-8073 was found to negatively regulate the gene and protein expression levels of these five targets. Finally, the administration of miR-8073 dramatically suppressed colorectal tumor growth in xenografted mice.

As the molecular function of microRNAs is to regulate target gene expression by binding the 3’-UTRs of target mRNAs, a number of microRNAs have been shown to control the proliferation and migration of cancer cells. The following examples have been reported: miR-1 inhibits cancer cell growth through the downregulation of oncogenes and/or transcription factors, including Met, HDAC4, PIM1, and Slug [9]; miR-21 induces myoblast apoptosis, which leads to cancer cachexia in patients with Toll-like receptor 7 [25]; miR-34a-5p controls cell proliferation through the modulation of the E2F signaling pathway [11]; miR-125a-3p suppresses cell proliferation and migration by targeting Fyn [12]; and miR-221-5p, which has the same seed sequence as miR-8073, inhibits cancer cell proliferation [14]. These microRNAs have been reported to be present at lower levels in cancer cells than in normal cells.

In our study, however, tumor suppressor or antiproliferative effects of the known disease markers miR-1, miR-34a-5p, and miR-125a-3p did not reach significant levels or were much lower than the effects of miR-8073. As their intracellular expression levels were similar in both cancer cells and normal cells, these microRNAs may be able to functionally regulate the cell cycle and cell proliferation only in normal cells. Even miR-221-5p, which is reported to elicit inhibitory effects on cell proliferation [14] and shares the same seed sequence as miR-8073, did not show similar effects to those of miR-8073. In our study, miR-8073 showed the most dramatic inhibition of cell proliferation in various cancer cells *in vitro* and suppressed colorectal tumor growth *in vivo*.

From the perspective of extracellular or exosomal microRNA analyses, several studies reported the association of miR-8073: its increased extracellular expression in acute myeloid leukemia cells [26], its participation in the regulation of the cardiometabolic phenotype [27], and its role as a serum biomarker of pancreatic cancer [3]. However, little was known about the molecular function of miR-8073, so we searched for target genes regulated by miR-8073.

Using microarrays, western blots, and luciferase reporter assays, potential targets of miR-8073 were identified: *FOXM1*, *CCND1*, *MBD3*, *KLK10*, and *CASP2*. These genes (and their encoded proteins) have been reported to be associated with the cell cycle, cell proliferation, and cancer development. Elevated levels of FOXM1, a transcription factor characterized by a DNA-binding domain called the Forkhead box [15], have been shown to be associated with poor survival in patients with solid tumors [16]. FOXM1 overexpression is involved in the early events of carcinogenesis [17] and plays an important role in cancer growth and metastasis [18]. Overexpression of CCND1 or cyclin D1, a well-known oncogene characterized by strong periodicity in protein levels throughout the cell cycle, is observed frequently in a variety of tumors. Besides miR-8073, various microRNAs have been reported to regulate CCND1; among them, miR-149 inhibits not only CCND1 but also FOXM1 [19]. Although the seed sequence similarity of miR-8073 and miR-149 was only 71%, these microRNAs may regulate a common signaling pathway that includes FOXM1/CCND1. KLK10 (kallikrein-10) is reportedly targeted by many microRNAs, such as let-7f, miR-224, and miR-516a, whose overexpression in ovarian cancer can lead to negative effects on cell proliferation [20]. MBD3 (methyl-CpG-binding domain protein-3) is known to induce pluripotent stem cells and may block the repression of reprogramming caused by the overexpression of miR-134 [28]. CASP2 (caspase-2) is reported to mediate tumor suppression as an initiator of survivin gene silencing [22]. In this study, caspase-2 was inhibited by miR-8073 overexpression; on the other hand, effector caspases-3 and -7 were activated, and apoptosis was induced. Therefore miR-8073 may induce apoptosis through the caspase-3/-7 cascade, which does not signal through caspase-2.

Although the five genes targeted by miR-8073 were all associated with cancer survival, it is unlikely that such dramatic tumor cell inhibition was caused by only one target. It is presumed that a combination of targets were involved, mutually amplifying the inhibitory effects. If so, miR-8073, a single entity that regulates multiple targets, may become a very effective therapeutic treatment for cancers of the colon and other organs.

## Conclusion

Based on a unique hypothesis and comprehensive screening of extracellular microRNAs, this study revealed for the first time that miR-8073 plays a previously unrecognized antiproliferative role in cancer by regulating multiple gene targets. Thus, miR-8073 holds promise as a potential cancer therapeutic treatment for colorectal cancer.

## Supporting information

S1 Fig Intracellular and extracellular expression of selected microRNAs in colorectal cancer cell HCT116 and normal cell HCoEpic. (TIF)

S2 Fig Intracellular and extracellular expression of miR-8073 in pancreatic cancer cell Panc-1. (TIF)

S3 Fig Viability of HCT116 cells for five days after transfection with miR-8073, negative control sequence (miR-NC), or mock. The star indicates p<0.05 in student’s t-test.
(TIF)

S4 Fig **Viability of HCT116 cells (A) and A549 cells (B) at fifth day from transfection with miR-1, miR-34a-5p, miR-125a-3p, miR-8073, or negative control sequence** (miR-NC). The star indicates p<0.05 in student’s t-test.
(TIF)

S5 Fig Expression of predicted mRNA targets was quantified by qRT-PCR in HCT116 cells treated with miR-8073 or negative control sequence. The star indicates p<0.05 in student’s t-test.
(TIF)

S6 Fig Relative luciferase activity of HEK293 cells co-transfected with either 3nM of miR-8073 mimic or microRNA negative control (miR-NC). With the luciferase reporter construct in which the 3’-UTR of each gene of interest was inserted. The control indicates the cells transfected with control vector, and the activity level of the control with miR-NC was set as 1.0. The error bars indicate the standard error of triplicate samples. The star indicates p<0.05 in student’s t-test.
(TIF)

S1 Table Gene list obtained from the mRNA expression analysis and the *in–silico* database analyses. (DOCX)

## References

[1] Ameres SL, Zamore PD., et al (2013) Diversifying microRNA sequence and function. Nat.Rev.Mol.Cell Biol. 14(8):475–88. 10.1038/nrm3611 23800994

[2] Friedman RC, Farh KK, Burge CB, Bartel DP., et al (2009) Most mammalian mRNAs are conserved targets of microRNAs. Genome Res. 19(1): 92–105. 10.1101/gr.082701.108 18955434 PMC2612969

[3] Kojima M, Sudo H, Kawauchi J, Takizawa S, Kondou S, Nobumasa H, Ochiai A., et al (2015) MicroRNA Markers for the Diagnosis of Pancreatic and Biliary-Tract Cancers. PLoS One 10(2):e0118220 10.1371/journal.pone.0118220 25706130 PMC4338196

[4] Shimomura A, Shiino S, Kawauchi J, Takizawa S, Sakamoto H, Matsuzaki J, Ono M, Takeshita F, Niida S, Shimizu C, Fujiwara Y, Kinoshita T, Tamura K, Ochiya T., et al (2016) Novel combination of serum microRNA for detecting breast cancer in the early stage. Cancer Sci. 107(3):326–34 10.1111/cas.12880 26749252 PMC4814263

[5] Hofsli E, Sjursen W, Prestvik WS, Johansen J, Rye M, Tranø G, Wasmuth HH, Hatlevoll I, Thommesen L., et al(2013) Identification of serum microRNA profiles in colon cancer. Br J Cancer 108(8):1712–9 10.1038/bjc.2013.121 23558896 PMC3668463

[6] Ogata-Kawata H, Izumiya M, Kurioka D, Honma Y, Yamada Y, Furuta K, Gunji T, Ohta H, Okamoto H, Sonoda H, Watanabe M, Nakagama H, Yokota J, Kohno T, Tsuchiya N., et al (2014) Circulating exosomal microRNAs as biomarkers of colon cancer. PLoS One. 9(4):e92921 10.1371/journal.pone.0092921 24705249 PMC3976275

[7] Al-Haidari AA, Syk I, Thorlacius H., et al(2017) MiR-155-5p positively regulates CCL17-induced colon cancer cell migration by targeting RhoA. Oncotarget. 8(9):14887–14896. 10.18632/oncotarget.14841 28146427 PMC5362452

[8] Armand-Labit Pradines A. et al, (2017) Circulating cell-free microRNAs as clinical cancer biomarkers. Biomol concepts 8(2):61–81 10.1515/bmc-2017-0002 Review. 28448269

[9] Han C, Shen JK, Hornicek FJ, Kan Q, Duan Z., et al (2017) Regulation of microRNA-1 (miR-1) expression in human cancer. Biochim Biophys Acta. 1860(2):227–232. 10.1016/j.bbagrm.2016.12.004 . 27923712

[10] Xu L, Xu Q, Li X, Zhang X., et al (2017) MicroRNA-21 regulates the proliferation and apoptosis of cervical cancer cells via tumor necrosis factor-α. Mol Med Rep. 2017 10;16(4):4659–4663. 10.3892/mmr.2017.7143 28765959 PMC5647022

[11] Tazawa H., Tsuchiya N, Izumiya M, Nakagama H., et al(2007) Tumor-suppresive miR-34a induces senescence-like growth arrest through modulation of the E2F pathway in human colon cancer cells. Proc Natl Acad Sci U S A. 104(39):15472–7. 10.1073/pnas.0707351104 17875987 PMC2000550

[12] Ninio-Many L, Grossman H, Shomron N, Chuderland D, Shalgi R., et al (2013) microRNA-125a-3p reduces cell proliferation and migration by targeting Fyn. J Cell Sci. 126 (13) 2867–76. 10.1242/jcs.123414 23606749

[13] Yoshioka Y, Konishi Y, Kosaka N, Katsuda T, Kato T, Ochiya T., et al (2013) Comparative marker analysis of extracellular vesicles in different human cancer types. J. Extracell. Vesicles. 2 10.3402/jev.v2i0.20424 24009892 PMC3760642

[14] Su A, He S, Tian B, Hu W, Zhang Z., et al(2013) MicroRNA-221 mediates the effects of PDGF-BB on migration, proliferation, and the epithelial-mesenchymal transition in pancreatic cancer cells. PLoS One. 8(8):e71309 10.1371/journal.pone.0071309 23967190 PMC3742757

[15] Wang Z, Ahmad A, Li Y, Banerjee S, Kong D, Sarkar FH., et al (2010) Forkhead box M1 transcription factor: a novel target for cancer therapy. Cancer Treat Rev. 36(2):151–6. 10.1016/j.ctrv.2009.11.006 20022709 PMC2838950

[16] Li L, Wu D, Yu Q, Li L, Wu P., et al (2017) Prognostic value of FOXM1 in solid tumors: a systematic review and meta-analysis. Oncotarget. 8(19):32298–32308. 10.18632/oncotarget.15764 Review. 28427178 PMC5458285

[17] Gemenetzidis E, Bose A, Riaz AM, Chaplin T, Young BD, Ali M, Sugden D, Thurlow JK, Cheong SC, Teo SH, Wan H, Waseem A, Parkinson EK, Fortune F, Teh MT., et al (2009) FOXM1 upregulation is an early event in human squamous cell carcinoma and it is enhanced by nicotine during malignant transformation PLoS One. 4(3): e4849 10.1371/journal.pone.0004849 19287496 PMC2654098

[18] Duan N, Hu X, Yang X, Cheng H, Zhang W., et al (2015) MicroRNA-370 directly targets FOXM1 to inhibit cell growth and metastasis in osteosarcoma cells. Int J Clin Exp Pathol 8(9):10250–60 26617733 PMC4637548

[19] Zhao L, Liu L, Dong Z, Xiong J., et al (2017) miR-149 suppresses human non-small cell lung cancer growth and metastasis by inhibiting the FOXM1/cyclin D1/MMP2 axis. Oncol Rep 38(6):3522–3530. 10.3892/or.2017.6047 29130108

[20] White NM, Chow TF, Mejia-Guerrero S, Diamandis M, Rofael Y, Faragalla H, Mankaruous M, Gabril M, Girgis A, Yousef GM., et al (2010) Three dysregulated miRNAs control kallikrein10 expression and cell proliferation in ovarian cancer. Br. J. Cancer. 102(8):1244–53 10.1038/sj.bjc.6605634 20354523 PMC2856011

[21] Li XQ, Guo YY, De W., et al (2012) DNA methylation and microRNAs in cancer. World J Gastroenterol. 18(9): 882–8. 10.3748/wjg.v18.i9.882 Review. 22408346 PMC3297046

[22] Guha M, Xia F, Raskett CM, Altieri DC., et al (2010) Caspase 2-mediated tumor suppression involves survivin gene silencing. Oncogene. 29(9):1280–92 10.1038/onc.2009.428 19935698 PMC2832727

[23] Kosaka N, Takeshita F, Yoshioka Y, Hagiwara K, Katsuda T, Ono M, Ochiya T., et al (2913) Exosomal tumor-suppressive microRNAs as novel cancer therapy: “exocure” is another choice for cancer treatment. Adv Drug Deliv Rev. 65(3):376–82. 10.1016/j.addr.2012.07.011 22841506

[24] Kosaka N, Iguchi H, Yoshioka Y, Takeshita F, Matsuki Y, Ochiya T., et al (2010) Secretory mechanisms and intercellular transfer of microRNAs in living cells. J Biol Chem. 285(23):17442–52. 10.1074/jbc.M110.107821 20353945 PMC2878508

[25] He wa, Calore F, Londhe P, Canella A, Guttridge DC, Croce CM, et al (2014) Microvesicles containing miRNAs promote muscle cell death in cancer cachexia via TLR7. Proc Natl Acad Sci U S A. 111(12):4525–9. 10.1073/pnas.1402714111 24616506 PMC3970508

[26] Horiguchi H, Kobune M, Kikuchi S, Yoshida M, Murata M, Murase K, Iyama S, Takada K, Sato T, Ono K, Hashimoto A, Tatekoshi A, Kamihara Y, Kawano Y, Miyanishi K, Sawada N, Kato J., et al (2016) Extracellular vesicle miR-7977 is involved in hematopoietic dysfunction of mesenchymal stromal cells via poly(rC) binding protein 1 reduction in myeloid neoplasms. Haematologica. 101(4) 437–47. 10.3324/haematol.2015.134932 26802051 PMC5004399

[27] Ghanbari M, Franco OH, de Looper HW, Hofman A, Erkeland SJ, Dehghan A., et al (2015) Genetic Variations in MicroRNA-Binding Sites Affect MicroRNA-Mediated Regulation of Several Genes Associated With Cardio-metabolic Phenotypes. Circ Cardiovasc Genet. 2015 6;8(3):473–86. 10.1161/CIRCGENETICS.114.000968 25814643

[28] Zhang L, Zheng Y, Sun Y, Zhang Y, Yan J, Chen Z, Jiang H., et al (2016) MiR-134-Mbd3 axis regulates the induction of pluripotency. J Cell Mol Med. (6):1150–8. 10.1111/jcmm.12805 26929159 PMC4882991

