# Supplementary figures and images for: MicroRNA-8073: Tumor suppressor and potential therapeutic treatment
Source: PLoS One. 2018 Dec 27;13(12):e0209750. doi: 10.1371/journal.pone.0209750 (PMC6307750; doi:10.1371/journal.pone.0209750)

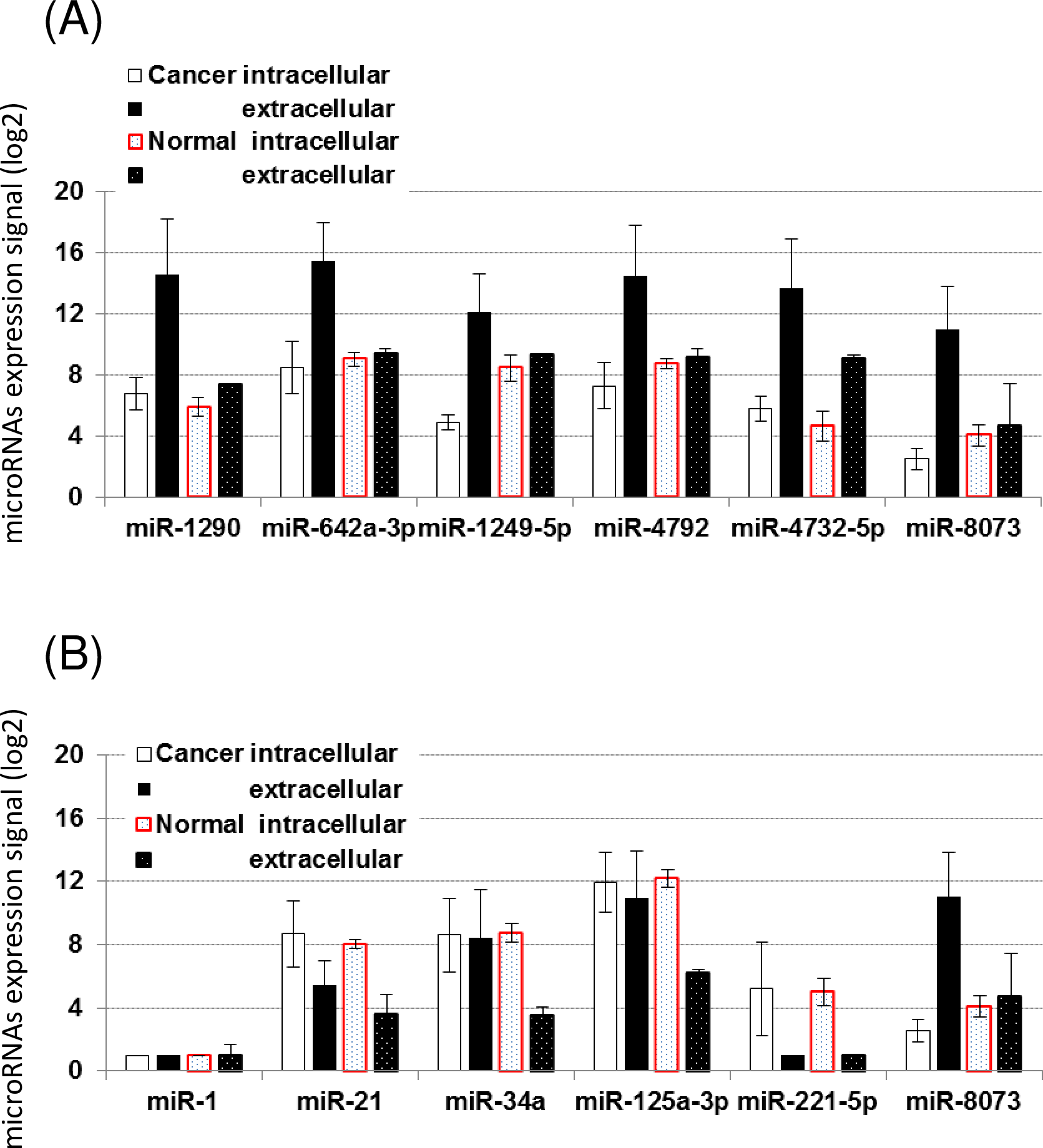

Supplement: S1 Fig — (TIF) [file pone.0209750.s001.tif]

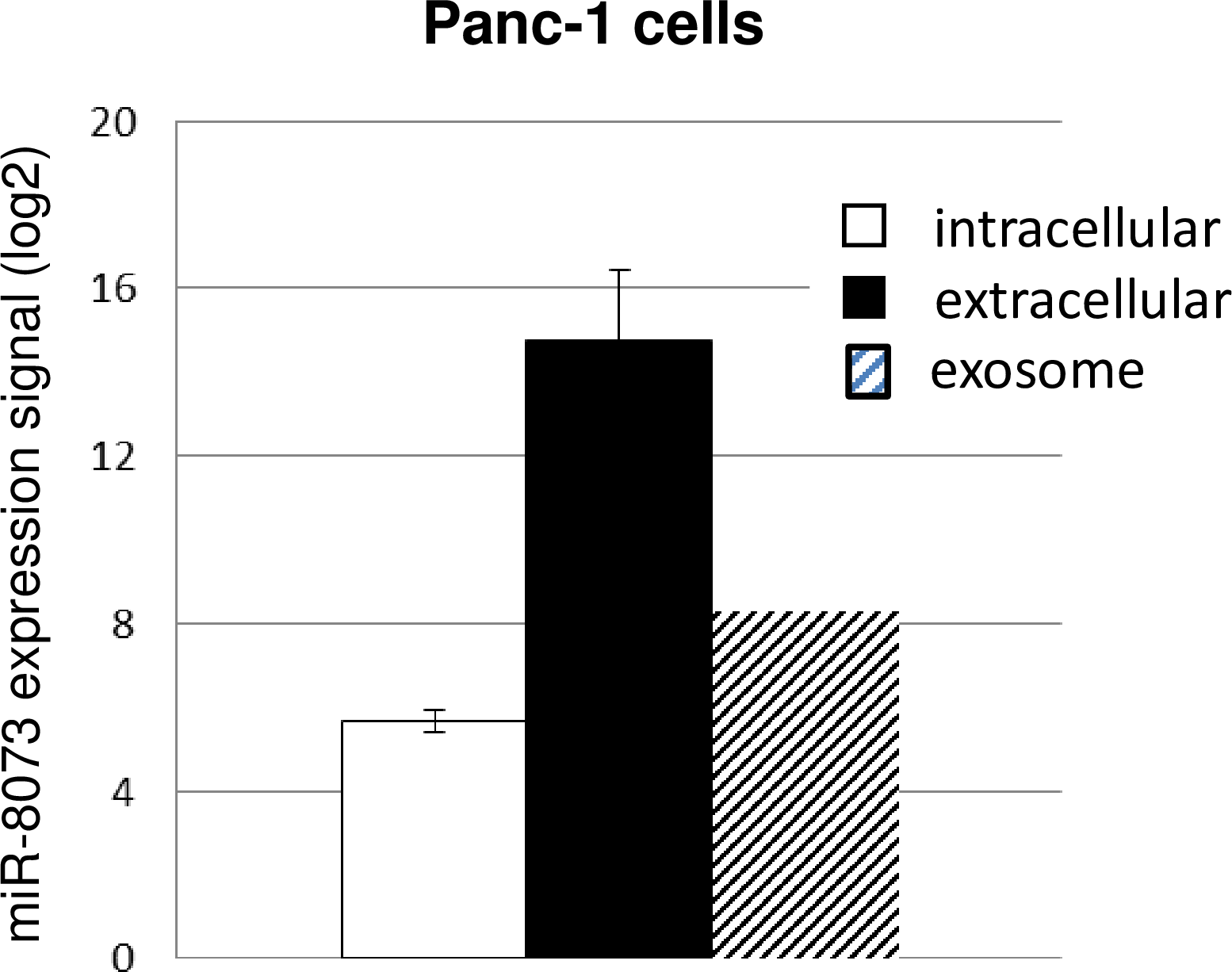

Supplement: S2 Fig — (TIF) [file pone.0209750.s002.tif]

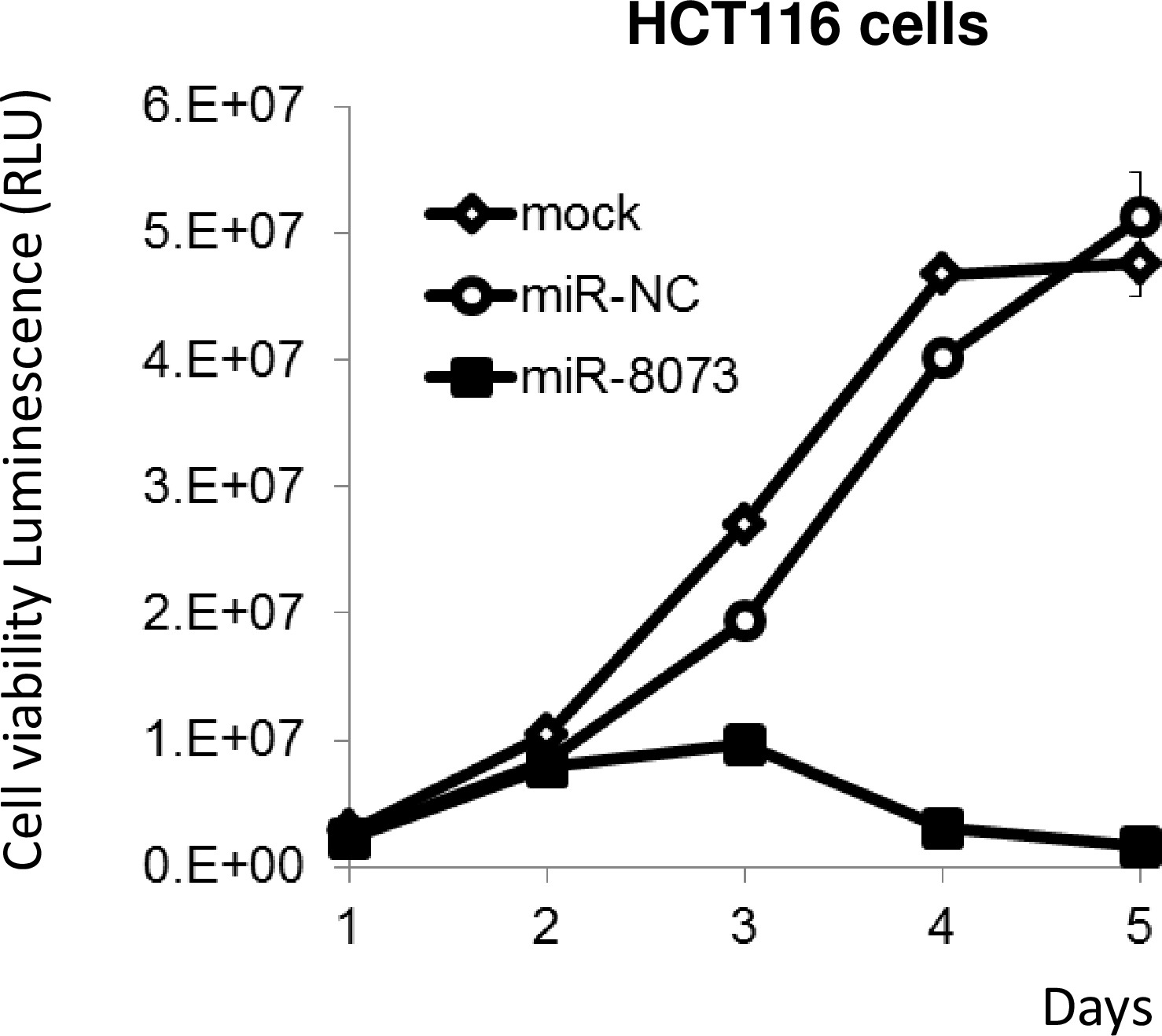

Supplement: S3 Fig — The star indicates p<0.05 in student’s t-test. (TIF) [file pone.0209750.s003.tif]

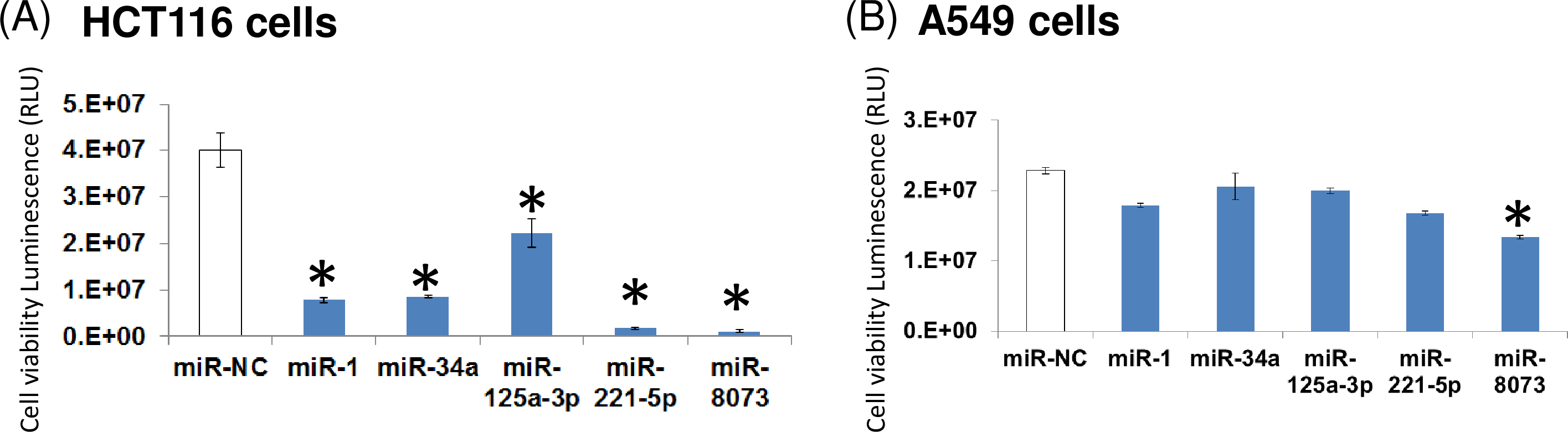

Supplement: S4 Fig — Viability of HCT116 cells (A) and A549 cells (B) at fifth day from transfection with miR-1, miR-34a-5p, miR-125a-3p, miR-8073, or negative control sequence (miR-NC). The star indicates p<0.05 in student’s t-test. (TIF) [file pone.0209750.s004.tif]

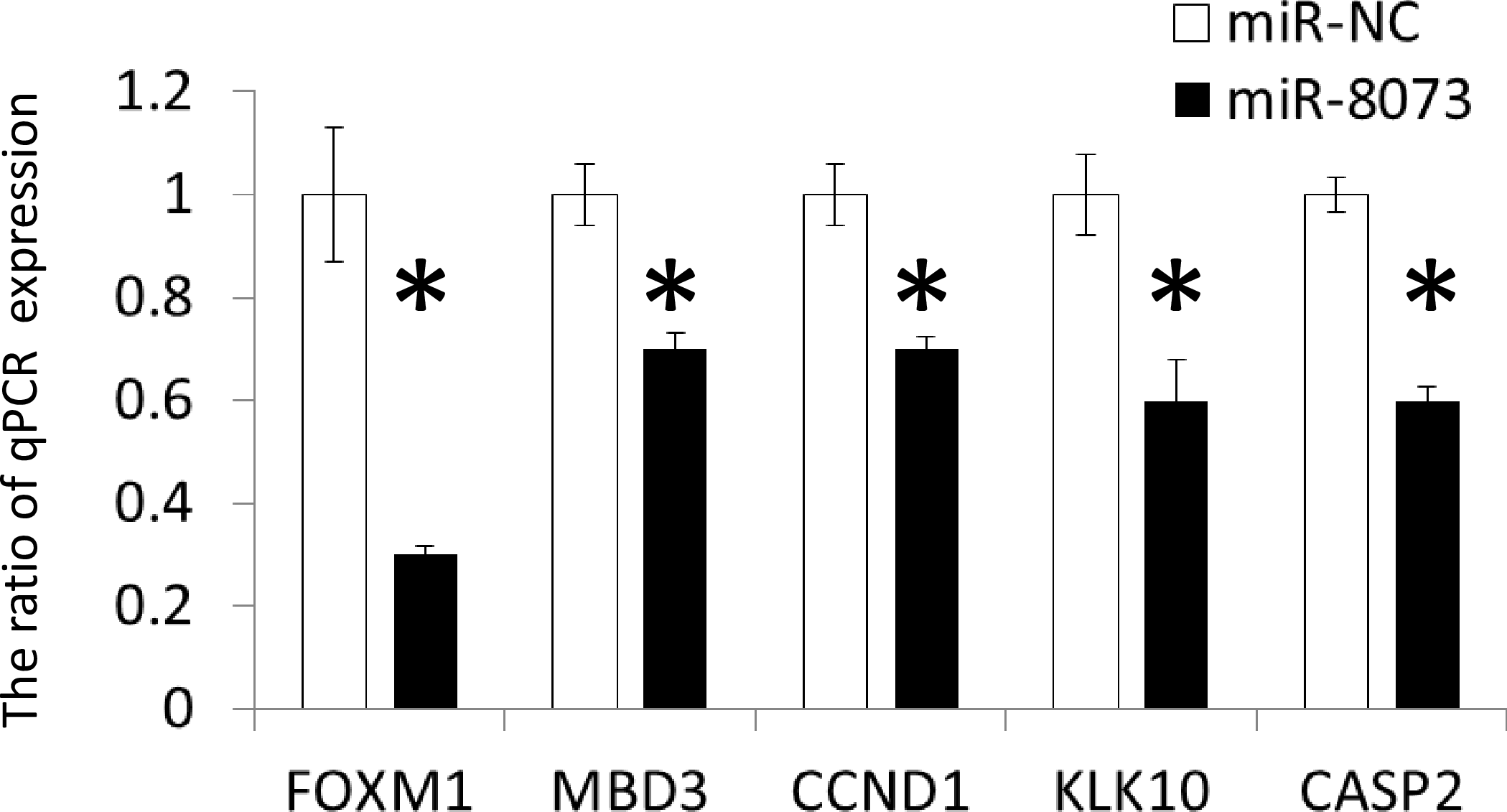

Supplement: S5 Fig — The star indicates p<0.05 in student’s t-test. (TIF) [file pone.0209750.s005.tif]

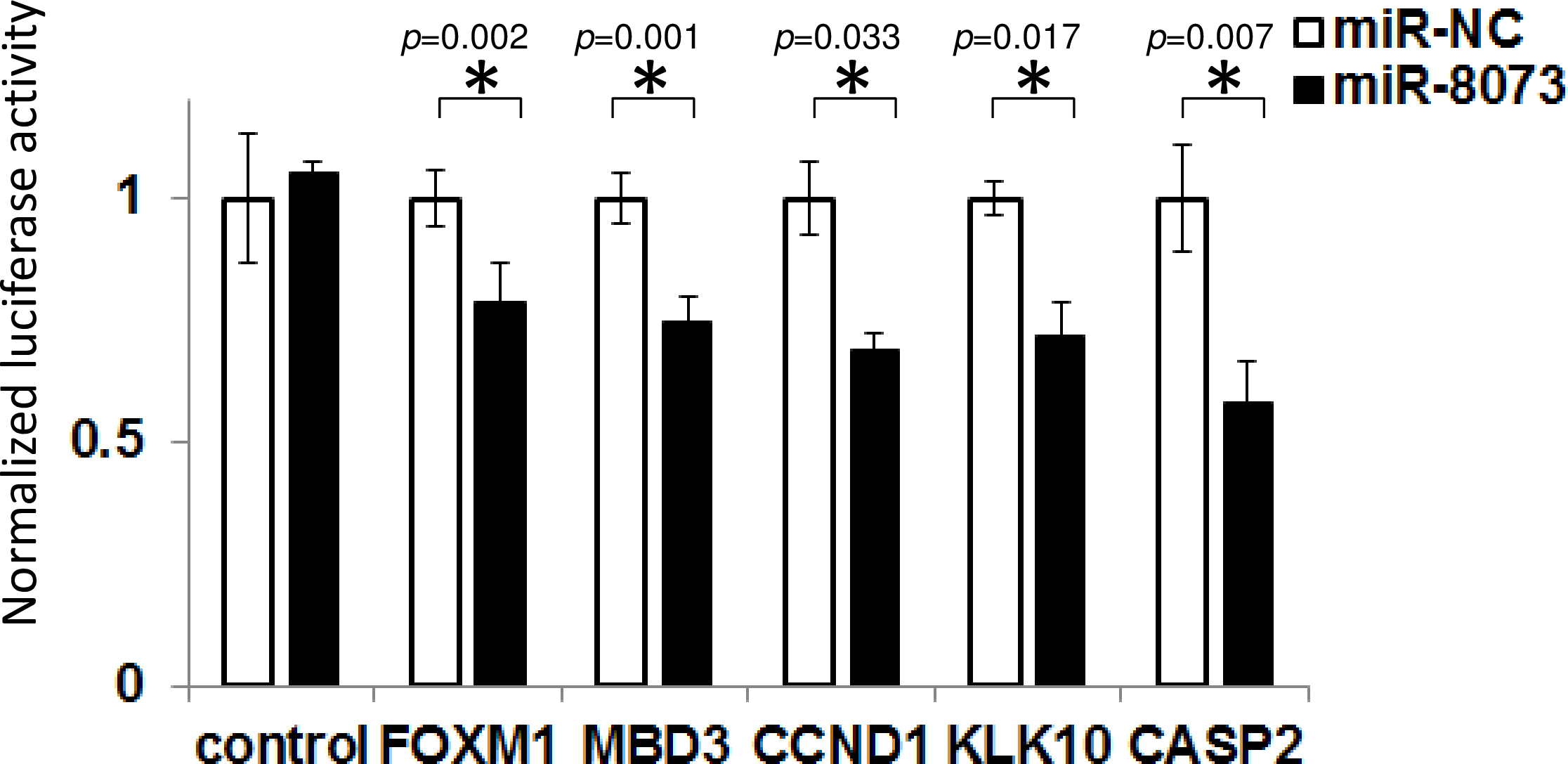

Supplement: S6 Fig — With the luciferase reporter construct in which the 3’-UTR of each gene of interest was inserted. The control indicates the cells transfected with control vector, and the activity level of the control with miR-NC was set as 1.0. The error bars indicate the standard error of triplicate samples. The star indicates p<0.05 in student’s t-test. (TIF) [file pone.0209750.s006.tif]
