# Supplementary material for: MicroRNA-8073: Tumor suppressor and potential therapeutic treatment
Source: PLoS One. 2018 Dec 27;13(12):e0209750. doi: 10.1371/journal.pone.0209750 (PMC6307750; doi:10.1371/journal.pone.0209750)
Supplement: S1 Table — (DOCX) [file pone.0209750.s007.docx]

SUPPORTING INFORMATION

| **mRNA expression analysis** | | | ***In-silico* database analyses** | | |
| --- | --- | --- | --- | --- | --- |
| **Gene** | **fold change(log2)** | **p.value** | **TargetScan**  **Total context++ score** | **microT**  **miTG score** | **miRDB**  **score** |
| FOXM1 | -3.23 | 4.58E-05 | -0.22 | - | - |
| TK1 | -2.84 | 4.25E-05 | - | - | - |
| DDX3Y | 2.59 | 9.48E-05 | - | - | - |
| HSF1 | -2.48 | 4.91E-05 | - | - | - |
| JAZF1 | 2.48 | 5.23E-05 | - | - | - |
| TSPAN13 | -2.42 | 5.89E-05 | - | - | - |
| ZDHHC18 | -2.33 | 8.83E-05 | -0.49 | 0.78 | 82 |
| PCYOX1L | -2.18 | 7.85E-05 | - | - | - |
| KANK2 | -2.13 | 0.000101 | - | - | - |
| CAPNS1 | -2.13 | 5.56E-05 | -0.2 | - | - |
| CERS2 | -2.03 | 8.18E-05 | - | - | - |
| KLK10 | -1.99 | 0.000255 | - | 0.73 | - |
| YOD1 | 1.95 | 0.000351 | - | - | - |
| CASP2 | -1.93 | 0.000374 | -0.87 | 0.99 | 84 |
| CTDSP2 | -1.92 | 0.000114 | - | 0.72 | - |
| TMEM109 | -1.87 | 0.000137 | -0.14 | 0.73 | 71 |
| C9orf40 | -1.86 | 0.000111 | - | - | - |
| CCND1 | -1.84 | 9.81E-05 | - | - | 79 |
| ANAPC15 | -1.84 | 0.000144 | -0.8 | - | 94 |
| ZDHHC12 | -1.83 | 0.00018 | - | - | - |
| BCL2L2-PABPN1 | -1.82 | 6.87E-05 | - | - | - |
| FAM64A | -1.80 | 0.000167 | - | - | - |
| FARSA | -1.78 | 0.000288 | - | - | - |
| CERS2 | -1.71 | 0.000164 | - | - | - |
| AC004066.2 | -1.70 | 0.000183 | - | - | - |
| SLC29A3 | -1.64 | 0.000343 | - | - | - |
| NINJ1 | -1.61 | 7.2E-05 | -0.23 | - | - |
| NA | -1.58 | 0.000203 | - | - | - |
| ZDHHC12 | -1.57 | 0.000239 | - | - | - |
| COPS7A | -1.57 | 0.000147 | -0.35 | - | - |
| MDC1 | -1.55 | 0.000141 | - | - | - |
| NKIRAS2 | -1.54 | 0.000285 | - | - | - |
| ARID3A | 1.53 | 0.000407 | - | - | - |
| LRP3 | -1.52 | 0.000206 | - | - | - |
| SIGIRR | -1.52 | 0.00016 | - | - | - |
| SNU13 | -1.52 | 0.000209 | - | - | - |
| KRTAP2-3 | 1.51 | 0.000417 | - | - | - |
| LAD1 | -1.49 | 0.000298 | -0.38 | - | - |
| PYCR1 | -1.48 | 0.000131 | - | - | - |
| MARCKSL1 | -1.41 | 0.000262 | - | - | - |
| EFNA4 | -1.39 | 0.000316 | 0 | - | - |
| ZCCHC9 | -1.37 | 0.000413 | -0.3 | - | - |
| ITPR3 | -1.34 | 0.000252 | - | - | - |
| JMJD8 | -1.34 | 0.00067 | -0.24 | - | - |
| THAP4 | -1.31 | 0.000226 | - | - | - |
| OGFRL1 | -1.29 | 0.000508 | - | - | - |
| TRAIP | -1.27 | 0.0006 | -0.2 | - | 84 |
| PCBD1 | -1.23 | 0.000384 | - | - | 53 |
| C8B | -1.22 | 0.00054 | - | - | - |
| CREBZF | 1.17 | 0.00062 | -0.14 | 0.92 | - |
| TPD52 | -1.16 | 0.000578 | -0.56 | 0.83 | 59 |
| ANXA5 | -1.15 | 0.000397 | - | - | - |
| C17orf62 | -1.12 | 0.000921 | -0.67 | - | - |
| INTS3 | -1.12 | 0.000474 | - | - | - |
| CYP24A1 | -1.09 | 0.000377 | - | - | - |
| UNG | -1.08 | 0.000908 | - | - | - |
| MBD3 | -1.05 | 0.000687 | -0.51 | - | - |
| FAM60A | -1.05 | 0.000778 | - | - | - |
| ANXA5 | -1.04 | 0.000684 | - | - | - |
| PRCC | -1.03 | 0.000996 | - | - | - |
